# Translation and cross-cultural adaptation of the Dubowitz Neurological Examination for premature infants in a high-risk outpatient clinic in Brazil

**DOI:** 10.1016/j.jped.2025.101460

**Published:** 2025-11-12

**Authors:** Ana Clara D. Massarollo, Guilherme W. Wendt, Lirane E.D. Ferreto, Ana P. Vieira, Gisele Arruda, Joseane R. da S. Nobre, Marina D. Massarollo, Débora M. Mazzo, Elizamara E.P. Segala, Claudicéia R. Pascotto, Franciele A.C. Follador

**Affiliations:** a Programa de Pós-Graduação em Ciências Aplicadas à Saúde (PPGCAS), Centro de Ciências da Saúde (CCS), Universidade Estadual do Oeste do Paraná (Unioeste), Francisco Beltrão, PR, Brazil; b Programa de Pós-Graduação: Residência em Pediatria, Universidade Estadual do Oeste do Paraná (Unioeste), Francisco Beltrão, Paraná, Brazil; c Centro de Ciências Biológicas e da Saúde (CCBS), Universidade Estadual do Oeste do Paraná (Unioeste), Cascavel, PR, Brazil; d Curso de Nutrição, Universidade Estadual do Oeste do Paraná (Unioeste), Francisco Beltrão, PR, Brazil; e Hospital Universitário Regional da Universidade Estadual de Ponta Grossa, Ponta Grossa, PR, Brazil

**Keywords:** Prematurity, Child development, Translation process, Assessment tool

## Abstract

**Objective:**

This study aimed to translate and cross-culturally adapt the Dubowitz Neurological Examination (DNE) instrument into Brazilian Portuguese for the neurological assessment of preterm newborns (PTNBs), as well as to evaluate its psychometric properties.

**Method:**

This is a methodological study of translation and cross-cultural adaptation. The methodological process consisted of two forward translations from the original to the target language, a synthesis of the translations, two back-translations, an evaluation by an expert committee, pre-testing, and the development and application of the final version in 40 PTNBs followed at a high-risk outpatient clinic.

**Results:**

Concordance among experts was 98.03 %, the intraclass correlation was 0.81, the content validity index (CVI) was 0.96 and the Kappa coefficient value was 0.76, indicating substantial agreement. Furthermore, the internal consistency indices were considered acceptable (α = 0.75). The comprehension of the 34 instrument items during the pre-test phase ranged from 82 % to 100 %.

**Conclusions:**

DNE was adequately translated and adapted for Brazilian culture, showing evidence of semantic, idiomatic, and conceptual equivalence. The results demonstrate satisfactory internal consistency and high inter-rater agreement.

## Introduction

Prematurity is characterized as birth before 37 weeks of gestational age (GA) [1] and is the leading cause of neonatal mortality [2]. It is a major global public health problem, affecting an estimated 15 million births annually, and is associated with increased risk of long-term neurodevelopmental sequelae, with profound impacts on children, families, and health systems worldwide [3, 4, 5, 6]. The relative risk of neurodevelopmental disability is higher in preterm infants compared to full-term infants [7]. Given this increased risk, it is of utmost importance to adopt measures such as public health policies, [8] focusing on the prevention of preterm births, parental counseling, the training of healthcare professionals, and the planning of care for preterm children within the healthcare system [7].

The use of clinical instruments for neurodevelopmental assessment in pediatrics is highly valuable in supporting the diagnosis of developmental delays, primarily to ensure that early interventions can be offered to children [9]. However, many of these instruments are expensive to acquire — for example, the complete Bayley Scales kit costs approximately one thousand dollars — and require specific training for their administration [10]. Furthermore, developing a new tool is highly challenging, as it requires years of research, clinical expertise, methodological rigor, and funding. In contrast, the translation, cross-cultural adaptation, and validation of an existing instrument, although a long process, is cost-effective and can save time and effort [11]. Cross-cultural adaptation is a complex process aimed at verifying linguistic accuracy and cultural appropriateness when preparing an instrument for use in a different context. These adaptations should be conducted across various settings, following rigorous methodological standards [12,13].

The Dubowitz Neurological Examination (DNE) (Supplementary material), originally developed in the United Kingdom in 1981 by Dubowitz et al. [14] and updated in 1998 [15] and 1999, [16] was designed to evaluate neurological function in preterm and term infants. The instrument demonstrated interobserver reliability and predictive validity for neurodevelopmental outcomes, [15,17] and has since been translated into several languages, including French, Dutch, Armenian, German, Greek, Brazilian Portuguese, and many others. However, not all of these translations followed a rigorous methodology for translation and cross-cultural adaptation.

Some neurological assessment instruments used in clinical practice have already undergone cross-cultural adaptation in several countries, such as the Hammersmith Infant Neurological Examination (HINE), [18, 19, 20] the Premie-Neuro Scale, [21] and the DNE, also known as the Hammersmith Neonatal Neurological Examination (HNNE) [22]. However, the latter was translated and adapted specifically for children at risk of cerebral palsy. Despite the existence of validated international tools, there is a scarcity of freely available and culturally adapted instruments in Brazil that can be applied in the routine follow-up of preterm newborns (PTNBs) within the Unified Health System (SUS). The availability of a Brazilian version of the DNE for preterm newborns can contribute to standardized, sensitive, and cost-effective clinical practices for neurodevelopmental screening, enabling both outpatient care and research comparability at the national and international levels.

Given the limited availability of free, easy-to-use, and time-efficient instruments for assessing high-risk PTNBs, and the importance of a Brazilian version for research and outpatient follow-up of this population, it was hypothesized that the Brazilian version of the DNE instrument would demonstrate adequate cultural and linguistic equivalence to the Brazilian context. Therefore, the present study aimed to translate, cross-culturally adapt, and evaluate the psychometric properties of the DNE instrument in Brazilian Portuguese, with the goal of its use in PTNBs treated at the general high-risk outpatient clinic of the Chronic Conditions Care Model (MACC) sector, within a health consortium in the micro-region of Francisco Beltrão, Paraná. The availability of a Brazilian version of the DNE may contribute to standardized, sensitive, and cost-effective clinical practices for the screening and monitoring of neurodevelopment in PTNBs within the context of the SUS.

## Methods

This is a methodological study involving translation and cross-cultural adaptation. The study was approved by the Research Ethics Committee under opinion number 3393,121.

### The instrument

The DNE assesses six dimensions: tone, type of tone, reflexes, movements, abnormal signs, and behavior, comprising a total of 34 items [16]. Each item is scored as abnormal (0 points), intermediate (0.5 points), or normal (1 point). Each dimension can be evaluated individually and has the following reference values: tone between 9 and 10; type of tone equal to 5; reflexes between 5 and 6; movements equal to 3; abnormal signs equal to 3; and behavior between 6 and 7 [15]. The test is considered normal when the total score ranges from 30.5 to 34 for full-term newborns, while the cutoff score for preterm infants is 26 [17].

### Translation and cross-cultural adaptation process

The DNE has already been translated and adapted twice into Brazilian Portuguese. Correr and Pfeifer performed a cross-cultural adaptation and reliability assessment in newborns at risk of cerebral palsy, [22] while a website that compiles translations of the instrument into several languages did not present a study with a rigorous translation methodology [23].

**Figure. 1:**
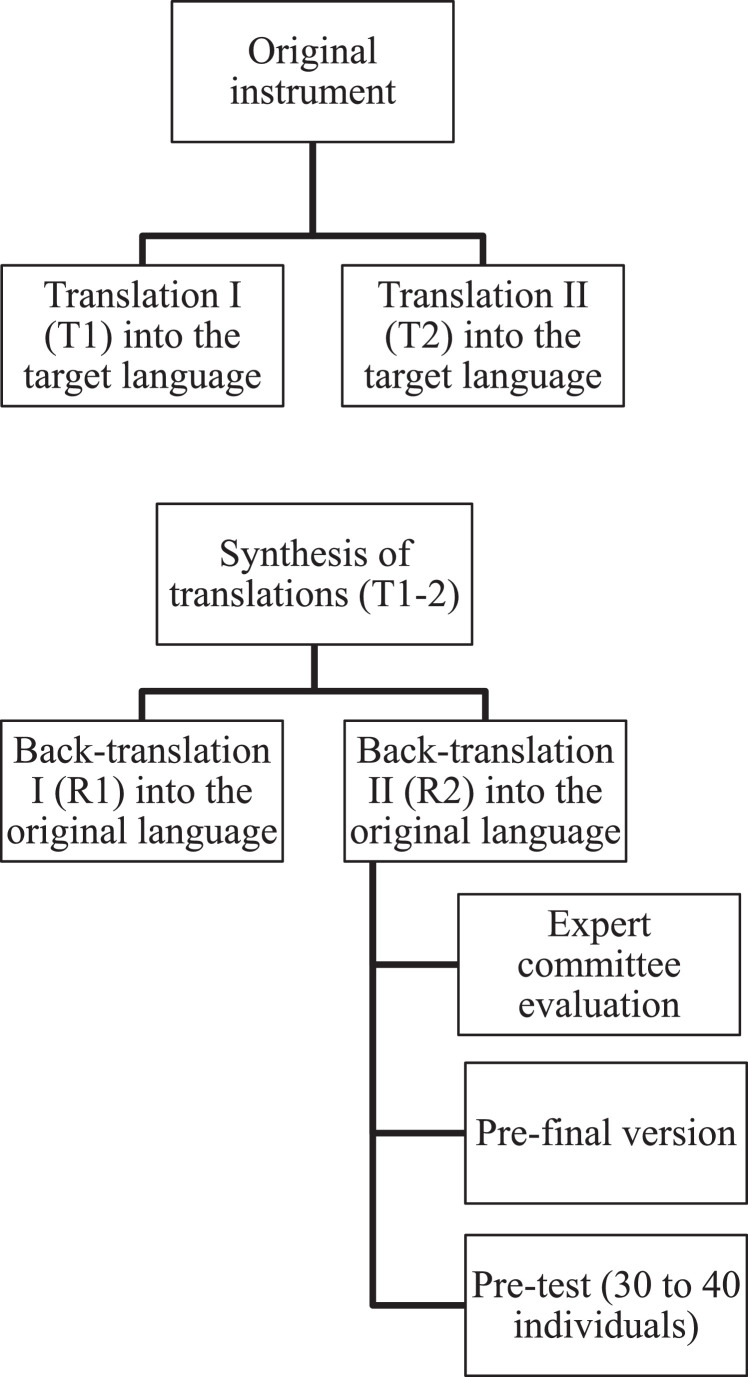
Methodological process for translation and cross-cultural adaptation.

For the translation process, the authors followed the methodological guidelines proposed by Beaton et al., [12] illustrated in Figure 1.

The first step consisted of two forward translations (T1 and T2) from the original language (English) into the target language (Brazilian Portuguese), carried out by native Portuguese speakers, one of whom had expertise in the field of neonatology. This stage allowed for the comparison of both translations. A synthesis was then created from these two versions, resulting in a single document (T-12), accompanied by a report detailing all identified discrepancies and their justifications.

Subsequently, two back-translations (R1 and R2) were performed by English language teachers who were blinded to the original instrument and worked exclusively from the T-12 version. Back-translation serves as one type of validity check, intended to identify major inconsistencies or conceptual errors in the translation. One translator was bilingual, with Portuguese as their native language and fluency in English, while the other was a native English speaker fluent in Portuguese. According to the recommendation by Beaton et al., [12] both translators should preferably be native speakers of the original instrument’s language; however, this composition was chosen due to the difficulty in finding a second available native English speaker.

Subsequently, all forward and back-translations, the synthesis, the report, and the original instrument were submitted to an expert committee composed of six members, including a methodologist, a physiotherapist, a pediatrician, and three translators who had participated in the previous stages of the process. The role of the expert committee was to consolidate all versions of the questionnaire and develop the pre-final version for field testing. After signing the Free and Informed Consent Form (FICF), the experts evaluated each item for semantic, linguistic, experiential, and conceptual equivalence using a judge committee review questionnaire. They indicated whether they fully agreed, partially agreed, or disagreed with each item, and were asked to specify the reasons for any disagreement.

Expert agreement was evaluated according to the criteria proposed by Polit and Beck, [24] who consider an agreement rate of 90 % or higher among committee members to be acceptable. Agreement was also assessed using the Kappa coefficient (K), which is interpreted according to five categories: values between 0.81 and 1.00 indicate almost perfect agreement; 0.61 to 0.80, substantial agreement; 0.41 to 0.60, moderate agreement; 0.21 to 0.40, fair agreement; and 0.00 to 0.20, slight to poor agreement [25].

The pretest phase of the new questionnaire aims to apply the pre-final version to subjects or patients from the target population. The pre-final version was administered by ten physiotherapists to 30 newborns aged up to six months. These professionals were chosen due to the ease of applying the instrument during routine child care; however, this configuration does not compromise its applicability to other professionals with expertise in the field. Each physiotherapist signed the FICF, as did the mother, father, or guardian of each infant. After administering the instrument, the physiotherapists completed a questionnaire evaluating the clarity and comprehensibility of the items, using a scale that ranged from 1 (strongly disagree) to 5 (strongly agree). Each item was required to achieve a minimum comprehension level of 80 % among the physiotherapists; if comprehension fell below 80 %, the item was to be reformulated [13,26]. Afterward, the Content Validity Index (CVI) was calculated according to the criteria established by Yusoff [27].

Subsequently, the final version was applied to 40 PTNBs who attended the public service High-Risk Outpatient Clinic of the Eighth Regional Health Department of Paraná, which covers 27 municipalities. The internal consistency of the scale and inter-rater agreement were evaluated using intraclass correlation. At this stage, internal consistency was assessed using Cronbach's alpha.

## Results

### Translation and cross-cultural adaptation

The present study demonstrated expert concordance of 98.03 %, with total agreement at 78.92 % and partial agreement at 19.01 %. The Kappa index was 0.76, indicating substantial agreement. Only one expert selected the "disagree" option for four items, which were subsequently revised by the expert committee, which considered the modifications appropriate for the pre-final version. The adjustments did not compromise the understanding of the items, being limited to synonym replacement, grammatical corrections, or punctuation changes. The CVI yielded an excellent value of 0.96.

In the pretest phase, comprehension of the 34 items ranged from 82 % to 100 %, exceeding the minimum established threshold. No cultural or conceptual modifications beyond linguistic adjustments were required.

### Psychometric properties

The internal consistency indices were considered acceptable (α = 0.75). Item-level values are presented in Table 1. Furthermore, the assessment of inter-rater agreement using the intraclass correlation coefficient (ICC) revealed a value of 0.81.

**Table 1:** Internal consistency indices of the scale per item.

Items	Α
q1 Posture	0.750
q2 Arm recoil	0.716
q3 Arm traction	0.748
q4 Leg recoil	0.754
q5 Leg traction	0.751
q6 Popliteal angle	0.745
q7 Head control 1 (Extensor tone)	0.736
q8 Head control 2 (Flexor tone)	0.720
q9 Head lag	0.755
q10 Ventral suspension	0.746
q11 Flexor tone (arms versus legs 1)	0.744
q12 Flexor tone (arms versus legs 2)	0.749
q13 Leg extensor tone	0.749
q14 Neck extensor tone (sitting)	0.744
q15 Increased extensor tone (horizontal)	0.745
q16 Tendon reflexes	0.739
q17 Gag/suck reflex	0.749
q18 Palmar grasp	0.744
q19 Plantar grasp	0.747
q20 Moro reflex	0.743
q21 Placing	0.745
q22 Spontaneous movements (quantitative)	0.749
q23 Spontaneous movements (qualitative)	0.744
q24 Head lift in prone position	0.736
q25 Abnormal posture of hands or toes	0.744
q26 Tremor	0.742
q27 Startle	0.742
q28 Eye movement	0.743
q29 Auditory orientation	0.749
q30 Visual orientation	0.729
q31 Attention	0.737
q32 Irritability	0.736
q33 Crying	0.721
q34 Consolability	0.756

Table 2 presents the characteristics of 40 PTNBs.

**Table 2:** Characteristics of 40 PTNBs.

Variables			
	Minimum	Maximum	Mean
Birthweght (grams)	775	3960	2194
Gestacional age (weeks)	25	36	34
APGAR score			
First minute	1	9	7,3
Fifth minute	5	10	8,7
Length of hospital stay (days)	0	86	23,1
	n ( %)		
Gender			
Male	16 (40 %)		
Female	24 (60 %)		
Classification of Prematurity			
Extremely premature	1 (2,5 %)		
Very premature	4 (10 %)		
Moderately premature	7 (17,5 %)		
Late preterm	28 (70 %)		
Type of delivery			
Cesarean section	27 (67,5 %)		
Vaginal birth	13 (32,5 %)		
Required Intensive Care Unit	4 (10 %)		
Required Intermediary Care Unit	11 (27,5 %)		
Required both Intensive and Intermediate CareUunit Required oxygen support	21 (52,5 %), 31 (77,5 %)		
Required Intubation	9 (22,5 %)		
Interferences of diagnosis	32 (80 %)		

## Discussion

This study aimed to translate and culturally adapt DNE into Brazilian Portuguese and to analyze its psychometric properties in preterm newborns. The results demonstrated substantial inter-rater agreement (Kappa = 0.76), high inter-rater reliability (ICC = 0.81), and acceptable internal consistency (α = 0.75), supporting the reliability of the adapted version.

The selection of the tool to be translated and culturally adapted was based on its ease of repeatability, as the examination can be completed in approximately 10 min. Additionally, the tool covers a variety of aspects of neurological function and can therefore provide a detailed profile of the neurological status of the assessed infant [26]. Furthermore, it is a well-tolerated assessment by newborns and shows inter-rater reliability greater than 96 % [16].

It was difficult to compare the results of the current study with the existing literature, as only one study involving the translation and adaptation of the same instrument (also known as HNNE) was found [22]. Therefore, this discussion will compare the results with studies that translated other neurological assessment instruments for children.

Similarly to the present study, Correr and Pfeifer [22] translated and adapted the Hammersmith Neonatal Neurological Examination (HNNE) into Portuguese and evaluated the instrument’s reliability for newborns at risk of cerebral palsy. These authors reported inter-rater agreement for the translated items above 80 % and satisfactory intraclass correlation coefficients (above 0.80), consistent with the findings of the current study.. However, when analyzing the internal consistency index values for individual items, all items in the present research showed significantly higher values. The highest value reported by Correr and Pfeifer [22] was α = 0.588 for the item “leg extensor tone,” compared to α = 0.749 for the same item in the current study, while their lowest value was α = 0.515 for the item “spontaneous movement (quantitative),” versus α = 0.749 in the present research. Additionally, the highest and lowest values in the current study were α = 0.756 and α = 0.721, respectively.

The HINE instrument is a neurological assessment tool used for typically developing and high-risk children between 2 and 24 months of age. It has also been translated and adapted into Turkish, Spanish and Portuguese, with ICC showing high values for the total score (ICC = 0.96; ICC = 0.98; ICC = 0.95, respectively) [18, 19, 20]. These findings are consistent with the present study, in which the ICC was 0.81. The Spanish version also showed substantial agreement (*k* = 0.61–0.80) for 10 out of 26 items, [19] which aligns with the present study, where the Kappa agreement between experts was 0.76.

In this sense, the Premie-Neuro Scale – a neurological examination for PTNBs – was translated into Spanish and validated. The analysis of internal consistency and reliability showed α = 0.677 and ICC = 0.783 for the total score, [21] which are lower values than those found in the present study. The authors justified this by pointing to the high variability in the movement subscale, due to the physiological state of PTNBs at the time of assessment [21]. In contrast, in the present study, when babies showed signs of fatigue, drowsiness, fever, or fear, the assessment was postponed until the situation was resolved before beginning or continuing data collection activities.

The cross-cultural adaptation of an instrument requires methodological rigor to ensure equivalence between the original and target languages. To achieve this, items must be not only accurately translated linguistically but also culturally adapted to maintain content validity [28].

Comprehension of the 34 items in the present study ranged from 82 % to 100 %, consistent with international validation studies of neurological and neonatal development instruments, which typically report comprehension rates around 80 % [29,30]. Discrepancies were observed in four items of the translated instrument; however, as reported by Furtado et al., [20] these were resolved through word substitutions, additions, and sentence reorganization, and in both studies, adaptations were made based on the suggestions of the experts who developed the pre-final version of the instruments. Nonetheless, the authors acknowledge that even items describing universal human behaviors may carry semantic or cultural nuances that require adaptation. In this study, no significant conceptual modifications were necessary, although such adjustments may be required in different cultural contexts.

The strengths of the present study include the adequate sample size in the pre-test phase and the fact that the participating physiotherapists and infants came from different institutions and municipalities, which provided greater diversity to the sample. As a limitation, it is important to note that one translator is bilingual, with Portuguese as their native language and fluency in English, while the other is a native English speaker fluent in Portuguese. Although Beaton et al. [8] recommend that, preferably, both translators should be native speakers of the original language. However, this composition was chosen due to the difficulty in finding a second available native speaker. Additionally, it is important to note that the pre-test was conducted exclusively with physical therapists, who may have greater technical familiarity with the instrument’s content. Therefore, future studies are recommended to assess the scale’s applicability across a broader range of healthcare professionals. The authors also emphasize that the results cannot be generalized to all neonatal populations, particularly to those with specific congenital or clinical conditions not represented in the sample.

The DNE was successfully translated and culturally adapted for Brazilian Portuguese, demonstrating semantic, idiomatic, and conceptual equivalence. The results show satisfactory internal consistency and high inter-rater agreement. For future studies, the authors suggest conducting a complete validation of the instrument with a representative sample and analyzing its psychometric properties, considering the number of items and population variability.

## Author contributions

**Ana Clara D. Massarollo:** Project design, Data collection, Original draft writing, Review and editing. **Guilherme W. Wendt:** Methodology, Data analysis, Writing – Review and editing. **Lirane E.D. Ferreto:** Methodology, Project administration – Review and editing. **Ana P. Vieira:** Project administration, Writing – Review and editing. **Gisele Arruda:** Writing – Review and editing. **Joseane R. da S. Nobre:** Writing – Review and editing. **Marina D. Massarollo:** Project administration, Writing – Review and editing. **Débora M. Mazzo:** Project design, Writing – Review and editing. **Elizamara E.P. Segala:** Writing – Review and editing. **Claudicéia R. Pascotto:** Writing – Review and editing. **Franciele A.C. Follador:** Project design, Methodology, Project administration, Writing –Review and editing.

## Conflicts of interest

The authors declare no conflicts of interest.

## Appendix Supplementary materials

Image, application 1

## Footnotes

Supplementary material associated with this article can be found in the online version at doi:10.1016/j.jped.2025.101460.

## References

[1] World Health Organization (WHO). WHO recommendations for care of the preterm or low birth weight infant. Geneva: World Health Organization; 2022. ISBN 978-92-4-005826-2.[Cited 2025 Aug 23] Available from: https://www.who.int/publications/i/item/9789240058262.

[2] Ohuma E.O., Moller A-B, Bradley E., Chakwera S., Hussian-Alkhateeb L., Lewin A. (2023). National, regional, and global estimates of preterm birth in 2020, with trends from 2010: a systematic analysis. Lancet.

[3] World Health Organization (WHO). Preterm Birth [Internet]. Geneva: World Health Organization; 2023 May 10. [Cited 2025 Aug 28] Available from: https://www.who.int/news-room/fact-sheets/detail/preterm-birth.

[4] Younge N., Goldstein R.F., Bann C.M., Hintz S.R., Patel R.M., Smith P.B. (2017). Survival and Neurodevelopmental Outcomes among Periviable Infants. N Engl J Med.

[5] Blencowe H., Cousens S., Chou D., Oestergaard M., Say L., Moller A.B. (2013). Born too soon: the global epidemiology of 15 million preterm births. Reprod Health.

[6] Moore T., Hennessy E.M., Myles J., Johnson S.J., Draper E.S., Costeloe K.L. (2012). Neurological and developmental outcome in extremely preterm children born in England in 1995 and 2006: the EPICure studies. BMJ.

[7] Mitha A., Chen R., Razaz N., Johansson S., Stephansson O., Altman M. (2024). Neurological development in children born moderately or late preterm: national cohort study. BMJ.

[8] Sadovsky A.D.I., Mascarello K.C., Miranda A.E., Silveira M.F. (2018). Socioeconomic inequality in preterm birth in four Brazilian birth cohort studies. J Pediatr (Rio J).

[9] Faruk T., King C., Muhit M., Islam M.K., Jahan I., Baset K.U. (2020). Screening tools for early identification of children with developmental delay in low- and middleincome countries: a systematic review. BMJ Open.

[10] Fernald LC, Kariger PK, Engle PL, Raikes A Examining early child development in low-income countries: a tool kit for the assessment of children in the first five years of life [Internet]. Washington (DC): The International Bank for Reconstruction and Development/The World Bank. 2009. [Cited 2025 Aug 24]. Available from: https://documents.worldbank.org/pt/publication/documents-reports/documentdetail/499021468332411850.

[11] Guillemin F. (1995). Cross-cultural Adaptation and Validation of Health Status Measures. Scand J Rheumatol.

[12] Beaton D.E., Bombardier C., Guillemin F., Ferraz M.B. (2000). Guidelines for the process of cross-cultural adaptation of self-report measures. Spine.

[13] Borsa J.C., Damásio B.F., Bandeira D.R. (2012). Cross-Cultural adaptation and validation of psychological instruments: some considerations. Paidéia.

[14] Dubowitz L.M., Dubowitz V. (1981). UK: Clinics in Developmental Medicine.

[15] Dubowitz L., Mercuri E., Dubowitz V. (1998). An optimality score for the neurologic examination of the term newborn. J Pediatr.

[16] Dubowitz L., Dubowitz V., Mercuri E. (1999). Neurological assessment of the preterm and full-term newborn infant.

[17] Grinaboldi A., Hinnig P., Moura S.P.S., MO G. (2015). Neurological assessment of newborn preterm infants: correlation with neonatal risk factors. Rev Neurocienc.

[18] Adigüzel H., Sarikabadayi U.Y., Apaydin U., Kirmaci Z.I.K., Gücüyener K., Karadeniz P.N. (2022). Turkish validity and reliability of the Hammersmith infant neurological examination (HINE) with high risk infant group: a preliminary study. Turk Arch Pediatr.

[19] Hidalgo-Robles Á, Merino-Andrés J., Rodríguez-Fernández A.L., Gutiérrez-Ortega M., León-Estrada I., Ródenas-Martínez M. (2024). Reliability, knowledge translation, and implementability of the Spanish version of the hammersmith infant neurological examination. Healthcare.

[20] Furtado M.A., Leite H.R., Klettenberg M.R., Rodrigues V.A., Ferreira L.S., Marques M.R. (2024). Translation and measurement properties of the Portuguese-Brazil version of the Hammersmith Infant Neurological Examination (HINE-Br). Rev Paul Pediatr.

[21] Fernández D., Alvarez M.J., Rodríguez D., Rodríguez M., Fernández E., Urdiales P. (2015). Spanish Validation of the Premie-Neuro Scale in Premature Infants. J Pediatr Nurs.

[22] Correr M.T., Pfeifer L.I. (2023). Cultural adaptation and reliability assessment of the Hammersmith neonatal neurological examination for Brazilian newborns at risk of cerebral palsy. Arq Neuropsiquiatr.

[23] Mac Keith Press (2025). https://www.mackeith.co.uk/hammersmith-neurological-examinations/hammersmith-neurological-examinations-subscriber-content/recording-and-scoring-proformas-and-guidance-notes-translations/.

[24] Polit D.F., Beck C.T. (2006). The Content Validity Index: are you sure you know what’s being reported? Critique and recommendations. Res Nurs Health.

[25] Landis J.R., Koch G.G. (1977). The measurement of observer agreement for categorical data. Biometrics.

[26] Dubowitz L., Ricci D., Mercuri E. (2005). The Dubowitz neurological examination of the full-term newborn. Ment Retard Dev Disabil Res Rev.

[27] Yusoff M.S. (2019). ABC of content validation and content validity index calculation. Educ Med J.

[28] Beaton D., Bombardier C., Guillemin F., Ferraz M.B. (2007).

[29] Rocha-Filho P.A., Hershey A.D. (2017). Pediatric Migraine Disability Assessment (PedMIDAS): translation into Brazilian Portuguese and cross-cultural adaptation. Headache.

[30] Piva E.K., Toso B.R., Carvalho A.R., Vieira C.S. (2018). Guimarães AT. Validation and categorization of the Parental Belief Scale of pre-term newborn babies. Acta Colomb Psicol.

