# Supplementary material for: Translation and cross-cultural adaptation of the Dubowitz Neurological Examination for premature infants in a high-risk outpatient clinic in Brazil
Source: J Pediatr (Rio J). 2025 Nov 12;101(6):101460. doi: 10.1016/j.jped.2025.101460 (PMC12662989; doi:10.1016/j.jped.2025.101460)

## Supplementary material

## Original Instrument - English

## Dubowitz Neurological Examination (DNE)

##
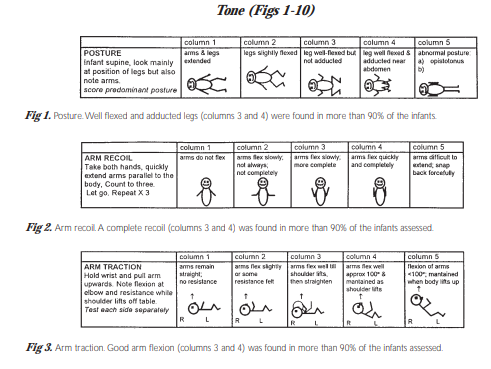


#
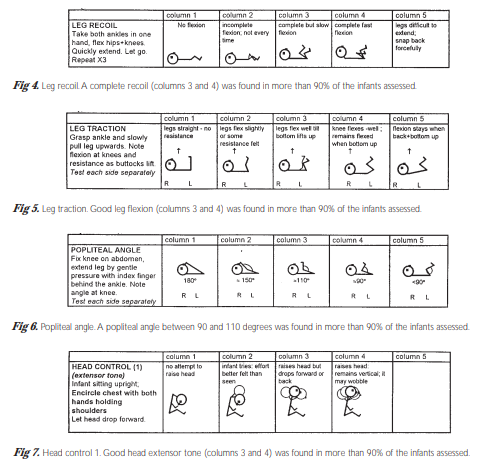


#
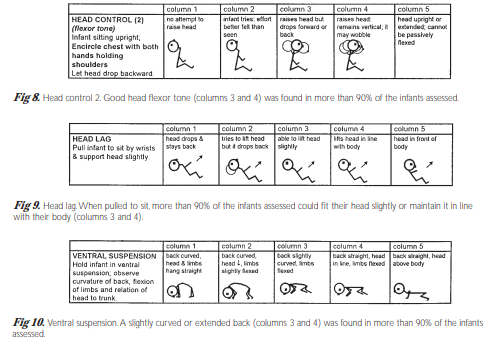


#
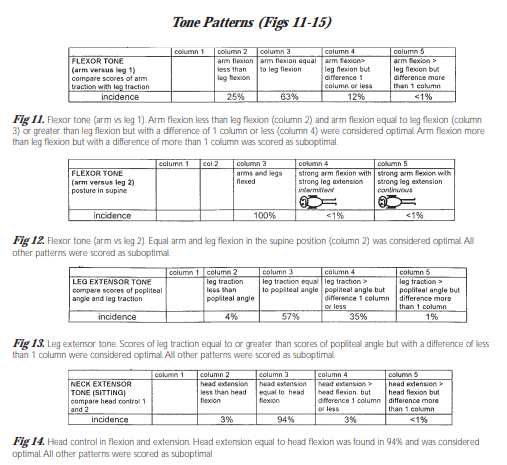


#
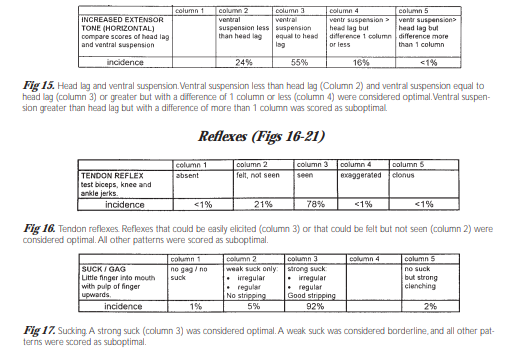


#
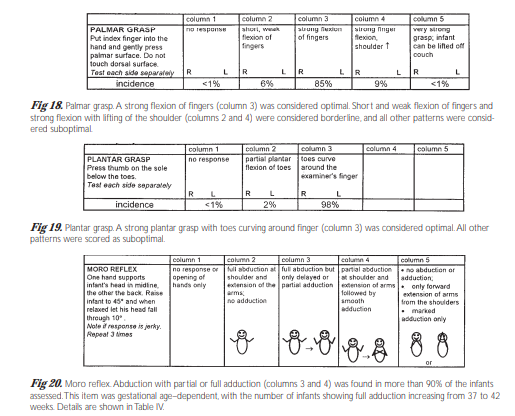


#
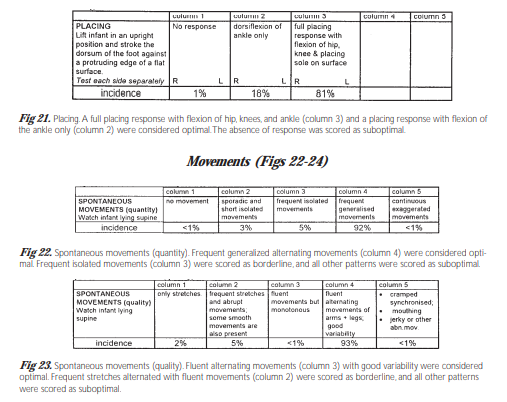


#
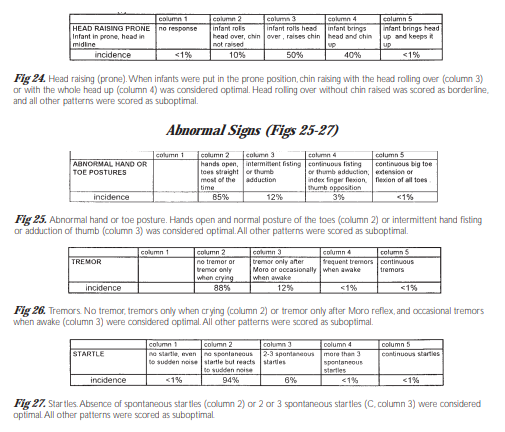


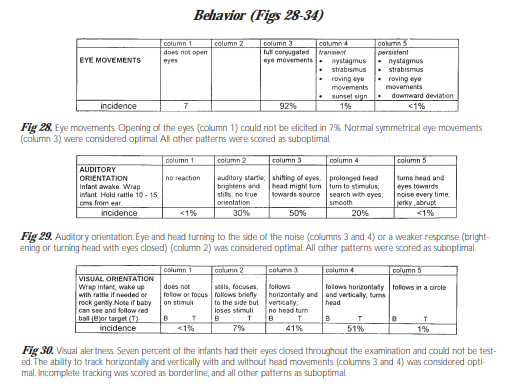


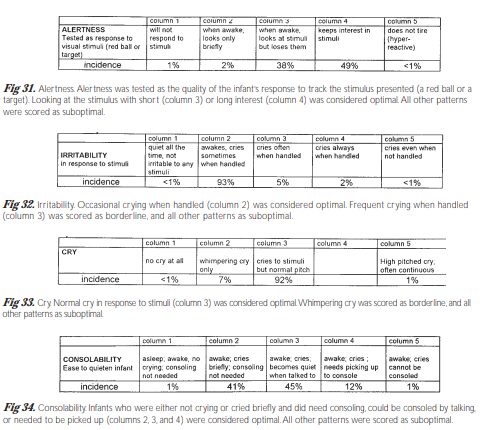


**Final version of the instrument – Portuguese**

**Exame Neurológico de Dubowitz**

| **Tônus (Figura 1-10)** | | | | | |
| --- | --- | --- | --- | --- | --- |
|  | Coluna 1 | Coluna 2 | Coluna 3 | Coluna 4 | Coluna 5 |
| Postura: Lactente em posição supina, observe principalmente a posição das pernas, mas também os braços. Pontue a postura predominante. | Braços e pernas estendidos  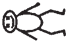 | Pernas levemente fletidas  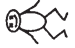 | Pernas bem fletidas, mas não aduzidas  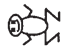 | Pernas bem fletidas e aduzidas próximas ao abdômen  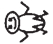 | Postura anormal  a) opistótono  b)  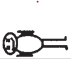 |
| **Figura 1** – Postura. Pernas bem fletidas e aduzidas (colunas 3 e 4) foram encontradas em mais de 90% dos lactentes. | | | | | |
|  | Coluna 1 | Coluna 2 | Coluna 3 | Coluna 4 | Coluna 5 |
| Retração do braço:  Pegue ambas as mãos do lactente, estenda rapidamente os braços paralelamente ao corpo. Conte até 3 e solte. Repita 3 vezes. | Braços não fletem  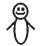 | Os braços fletem lentamente, não todas as vezes, não completamente  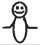 | Braços fletem lentamente, de forma mais completa  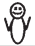 | Braços fletem rápida e completamente  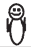 | Dificuldade na extensão dos braços, retorno instantâneo e vigoroso |
| **Figura 2** – Recuo do braço. Um recuo completo (colunas 3 e 4) foi encontrado em mais de 90% dos lactentes avaliados. | | | | | |
|  | Coluna 1 | Coluna 2 | Coluna 3 | Coluna 4 | Coluna 5 |
| Tração do braço: Segure o punho e puxe o braço para cima. Observe a flexão do cotovelo e a resistência enquanto o ombro deixa a bancada. Teste cada lado separadamente. | Braços permanecem estendidos, sem resistência  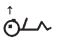  D E | Braços fletem levemente ou alguma resistência é sentida  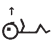  D E | Braços bem fletidos até os ombros levantarem, então se estendem  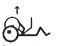  D E | Braços bem fletidos a aproximadamente 100^o^ e mantidos com a elevação dos ombros  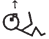  D E | Flexão dos braços <100^o^, mantidos quando o corpo é levantado para cima  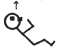  D E |
| **Figura 3** – Tração do braço. Boa flexão do braço (colunas 3 e 4) foi encontrada em mais de 90% dos lactentes avaliados. | | | | | |
|  | Coluna 1 | Coluna 2 | Coluna 3 | Coluna 4 | Coluna 5 |
| Retração da perna: Pegue ambos os tornozelos em uma mão e flexione quadris + joelhos. Rapidamente estenda e solte. Repita 3x | Sem flexão  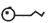 | Flexão incompleta, não todas as vezes  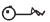 | Flexão completa, mas lenta  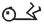 | Flexão rápida e completa  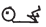 | Dificuldade para estender as pernas; retorno instantâneo e vigoroso. |
| **Figura 4** – O completo recuo das pernas (colunas 3 e 4) é encontrado em mais de 90% dos lactentes avaliados. | | | | | |
|  | Coluna 1 | Coluna 2 | Coluna 3 | Coluna 4 | Coluna 5 |
| Tração da perna: Segure o tornozelo e puxe lentamente a perna para cima. Observe a flexão dos joelhos e sua resistência à medida que o quadril se eleva. Teste cada lado separadamente | Pernas retas, sem resistência  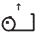  D E | Pernas fletem levemente ou alguma resistência é sentida  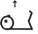  D E | Pernas bem fletidas até que o quadril se eleve  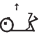  D E | O joelho flete bem e permanece fletido quando o quadril se eleva  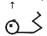  D E | Flexão permanece quando costas e quadril se elevam  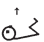  D E |
| **Figura 5** – Tração da perna. Boa flexão da perna (colunas 3 e 4) foi encontrada em mais de 90% dos lactentes avaliados. | | | | | |
|  | Coluna 1 | Coluna 2 | Coluna 3 | Coluna 4 | Coluna 5 |
| Ângulo poplíteo: Posicione e mantenha o joelho sobre o abdômen e estenda a perna com uma pressão suave com o dedo indicador atrás do tornozelo. Observe o ângulo do joelho. Teste cada lado separadamente | 180^o^  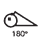  D E | ≈150^o^  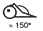  D E | ≈ 110^0^  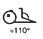  D E | ≈ 90^0^  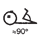  D E | <90^o^  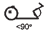  D E |
| **Figura 6** – Ângulo poplíteo. Um ângulo poplíteo entre 90 e 110 graus foi encontrado em mais de 90% dos lactentes avaliados. | | | | | |
|  | Coluna 1 | Coluna 2 | Coluna 3 | Coluna 4 | Coluna 5 |
| Controle de cabeça 1 (tônus extensor): Com o lactente sentado em posição vertical, envolva o tórax com ambas as mãos segurando os ombros. Deixe a cabeça cair para frente | Não há tentativa de levantar a cabeça  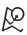 | Lactente tenta elevar a cabeça, mas não consegue. Esforço melhor sentido que visto  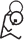 | Lactente eleva a cabeça, porém esta cai para frente ou para trás  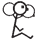 | Lactente eleva a cabeça, mantém na vertical, porém oscila  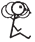 |  |
| **Figura7** – Controle de cabeça 1. Bom tônus extensor de cabeça (colunas 3 e 4) foi encontrado em mais de 90% dos lactentes avaliados | | | | | |
|  | Coluna 1 | Coluna 2 | Coluna 3 | Coluna 4 | Coluna 5 |
| Controle de cabeça 2 (tônus flexor): Lactente sentado na posição vertical. Envolva o tórax com ambas as mãos e segure os ombros. Deixe a cabeça cair para trás | Não há tentativa de levantar a cabeça  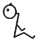 | Lactente tenta elevar a cabeça, esforço melhor sentido do que visto  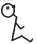 | Levanta a cabeça, porém esta cai para frente ou para trás  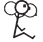 | Levanta a cabeça, permanece vertical. Ela pode oscilar  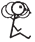 | Cabeça para cima ou estendida: não pode ser fletida passivamente |
| **Figura 8** – Controle de cabeça 2. Bom tônus flexor da cabeça (colunas 3 e 4) foi encontrado em mais de 90% dos lactentes avaliados. | | | | | |
|  | Coluna 1 | Coluna 2 | Coluna 3 | Coluna 4 | Coluna 5 |
| Atraso da cabeça: Puxe o lactente para a posição sentada pelos punhos e apoie a cabeça levemente. | Cabeça cai e permanece para trás  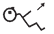 | Tenta levantar a cabeça, mas ela cai para trás  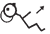 | É capaz de elevar a cabeça levemente  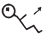 | Eleva a cabeça alinhada com o corpo  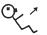 | Cabeça à frente do corpo  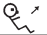 |
| **Figura 9** – Atraso da cabeça. Quando puxado para sentar, mais de 90% dos lactentes avaliados podiam elevar a cabeça levemente ou mantê-la alinhada com seu corpo (colunas 3 e 4). | | | | | |
|  | Coluna 1 | Coluna 2 | Coluna 3 | Coluna 4 | Coluna 5 |
| Suspensão ventral: Segure o lactente em suspensão ventral, observe a curvatura da coluna, flexão dos membros e a relação da cabeça com o tronco | Coluna curva, cabeça e membros ficam pendentes e retos  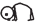 | Coluna curva, cabeça membros levemente fletidos  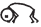 | Coluna levemente curvada, membros fletidos  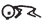 | Coluna ereta, cabeça alinhada, membros fletidos  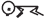 | Coluna ereta cabeça acima do corpo  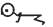 |
| **Figura 10** – Suspensão ventral. Uma leve curvatura ou coluna estendida (colunas 3 e 4) foi encontrada em mais de 90% dos lactentes avaliados.  **Padrões do tônus (Figura 11-15)**   \|  \| Coluna 1 \| \| Coluna 2 \| Coluna 3 \| Coluna 4 \| Coluna 5 \| \| --- \| --- \| --- \| --- \| --- \| --- \| --- \| \| Tônus flexor (braços versus pernas 1): Compare a pontuação da tração do braço com a tração da perna \|  \| \| Flexão de braço menor que a flexão da perna \| Flexão de braço igual a flexão de perna \| Flexão de braço > que flexão de perna, mas diferem uma coluna ou menos \| Flexão de braço > que flexão de perna, mas diferem mais que uma coluna \| \| Incidência \|  \| \| 25% \| 63% \| 12% \| <1% \| \| **Figura 11** – Tônus flexor (braços x pernas 1). Flexão do braço menor que a flexão da perna (coluna 2)e flexão do braço igual flexão da perna (coluna 3) ou maior que flexão da perna, mas com diferença de uma coluna ou menos (coluna 4) foram consideradas ótimas. Flexão do braço maior que flexão da perna, mas com diferença de mais de uma coluna foi considerado subótimo. \| \| \| \| \| \| \| \|  \| Coluna 1 \| \| Coluna 2 \| Coluna 3 \| Coluna 4 \| Coluna 5 \| \| Tônus flexor (braços versus pernas 2): Posição supina \|  \| \|  \| Braços e pernas fletidos \| Forte flexão do braço e forte extensão intermitente da perna  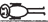 \| Forte flexão do braço e forte extensão contínua da perna  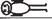 \| \| Incidência \|  \| \|  \| 100% \| <1% \| <1% \| \| **Figura 12** – Tônus flexor (braços x pernas 2). Flexão de braços e pernas iguais na posição supina (coluna 3) foi considerada ótima. Todos os outros padrões foram classificados como subótimos. \| \| \| \| \| \| \| \|  \| Coluna 1 \| \| Coluna 2 \| Coluna 3 \| Coluna 4 \| Coluna 5 \| \| Tônus extensor da perna: Compare a pontuação do ângulo poplíteo e da tração da perna \|  \| \| Tração da perna menor que o ângulo poplíteo \| Tração da perna igual ao ângulo poplíteo \| Tração da perna > que ângulo poplíteo, mas diferem 1 coluna ou menos \| Tração da perna > que ângulo poplíteo, mas diferem mais de 1 coluna \| \| Incidência \|  \| \| 4% \| 57% \| 35% \| 1% \| \| **Figura 13** – Tônus extensor da perna. Classificação da tração de perna igual ou maior que a classificação do ângulo poplíteo, mas com a diferença de menos de uma coluna foram considerados ótimos. Todos os outros padrões foram classificados como subótimos. \| \| \| \| \| \| \| \|  \| Coluna 1 \| \| Coluna 2 \| Coluna 3 \| Coluna 4 \| Coluna 5 \| \| Tônus extensor do pescoço (sentado): Compare controle de cabeça 1 e 2 \|  \| \| Extensão de cabeça menor que flexão de cabeça \| Extensão da cabeça igual a flexão de cabeça \| Extensão da cabeça > que flexão da cabeça, mas diferem 1 coluna ou menos \| Extensão da cabeça > que flexão da cabeça, mas diferem mais de 1 coluna \| \| Incidência \|  \| \| 3% \| 94% \| 3% \| <1% \| \| **Figura 14** – Controle de cabeça em flexão e extensão. Extensão de cabeça igual a flexão de cabeça foi encontrada em 94% e foi considerada ótima. Todos os outros padrões foram classificados como subótimos. \| \| \| \| \| \| \| \|  \| Coluna 1 \| \| Coluna 2 \| Coluna 3 \| Coluna 4 \| Coluna 5 \| \| Tônus extensor aumentado (horizontal): Compare a pontuação do atraso da cabeça e suspensão ventral \|  \| \| Suspensão ventral menor que o atraso da cabeça \| Suspensão ventral equivale ao atraso da cabeça \| Suspensão ventral > que o atraso da cabeça, mas diferem 1 coluna ou menos \| Suspensão ventral > que o atraso da cabeça, mas diferem mais de 1 coluna \| \| Incidência \|  \| \| 24% \| 55% \| 16% \| <1% \| \| **Figura 15** – Atraso da cabeça e suspensão ventral. Suspensão ventral menor que a queda da cabeça (coluna 2) e suspensão ventral igual ao atraso da cabeça (coluna 3) ou maior, mas com uma diferença de uma coluna ou menos (coluna 4)foram considerados ótimos. Suspensão ventral maior do que atraso da cabeça, mas com uma diferença de mais de uma coluna foi classificado como subótima.  **Reflexos (Figura 16-21)** \| \| \| \| \| \| \| \|  \| Coluna 1 \| \| Coluna 2 \| Coluna 3 \| Coluna 4 \| Coluna 5 \| \| Reflexos tendinosos: Biciptal, patelar (joelho), Aquileu (tornozelo) \| Ausente \| \| Sentido, porém não visualizado \| Visualizado \| Exagerado \| Clônus \| \| Incidência \| <1% \| \| 21% \| 78% \| <1% \| <1% \| \| **Figura 16** – Reflexos tendinosos. Reflexos que podem ser facilmente provocados (coluna 3) ou que podem ser sentidos, mas não visualizados (coluna 2) foram considerados ótimos. Todos os outros padrões foram classificados como subótimos. \| \| \| \| \| \| \| \|  \| Coluna 1 \| \| Coluna 2 \| Coluna 3 \| Coluna 4 \| Coluna 5 \| \| Reflexo de engasgo/sucção: Inserir o dedo mínimo na boca do lactente com a polpa do dedo voltada para cima \| Sem reflexo de engasgo, sem sucção \| \| Somente uma fraca sucção irregular ou regular. Sem ordenha \| Sucção forte, irregular ou regular. Boa ordenha \|  \| Sem sucção, mas forte aperto (pega) \| \| Incidência \| 1% \| \| 5% \| 92% \|  \| 2% \| \| **Figura 17** – Sucção. Uma sucção forte (coluna 3) foi considerada ótima. Uma sucção fraca foi considerada limítrofe, e todos os outros padrões foram considerados como subótimos. \| \| \| \| \| \| \| \|  \| Coluna 1 \| \| Coluna 2 \| Coluna 3 \| Coluna 4 \| Coluna 5 \| \| Preensão palmar: O examinador posiciona o dedo indicador na face palmar da mão do lactente e pressiona levemente. Não toque a face dorsal da mão. Teste cada lado separadamente \| Sem resposta  D E \| \| Flexão fraca e curta dos dedos  D E \| Forte flexão dos dedos  D E \| Flexão forte dos dedos, ombros se elevam  D E \| Preensão muito forte, lactente pode ser levantado da maca  D E \| \| Incidência \| <1% \| \| 6% \| 85% \| 9% \| <1% \| \| **Figura 18** – Preensão palmar. Uma flexão forte dos dedos (coluna 3) foi considerada ótima. Flexão fraca e curta dos dedos e Flexão forte dos dedos, ombros se elevam (colunas 2 e 4) foram consideradas limítrofes, e todos os outros padrões foram classificados como subótimos. \| \| \| \| \| \| \| \|  \| Coluna 1 \| \| Coluna 2 \| Coluna 3 \| Coluna 4 \| Coluna 5 \| \| Preensão plantar: Pressione o polegar na planta do pé, logo abaixo dos dedos. Teste cada lado separadamente \| Sem resposta  D E \| \| Flexão plantar parcial dos dedos  D E \| Os dedos dobram-se ao redor do dedo do examinador  D E \|  \|  \| \| Incidência \| <1% \| \| 2% \| 98% \|  \|  \| \| **Figura 19** – Preensão plantar. Uma forte preensão plantar com os dedos dos pés do lactente curvando-se ao redor do polegar do examinador (coluna 3) foi considerada ótima. Todos os outros padrões foram classificados como subótimos. \| \| \| \| \| \| \| \|  \| Coluna 1 \| \| Coluna 2 \| Coluna 3 \| Coluna 4 \| Coluna 5 \| \| Reflexo de Moro: Uma mão apoia a cabeça na linha média e a outra o tronco. Eleve o lactente até 45º e quando estiver relaxado, deixe a cabeça e tronco cair até 10º. Observe se a resposta é espasmódica. Repita 3x \| Sem resposta ou apenas abre as mãos \| \| Abdução total dos ombros e extensão dos braços, sem adução  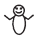 \| Abdução total, mas adução atrasada ou parcial  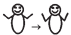 \| Abdução parcial dos ombros e extensão dos braços seguida de uma suave adução  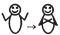 \| Nenhuma abdução ou adução, somente extensão dos braços a partir dos ombros ou somente adução acentuada  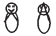  ou \| \| **Figura 20** – Reflexo de Moro. Abdução com total ou parcial adução (colunas 3 e 4) foi encontrada em mais de 90% dos lactentes avaliados. Esse item é dependente da idade gestacional, com um número de lactentes mostrando aumento na adução total entre 37 a 42 semanas. \| \| \| \| \| \| \| \|  \| Coluna 1 \| \| Coluna 2 \| Coluna 3 \| Coluna 4 \| Coluna 5 \| \| Colocação: Eleve o lactente na posição vertical e toque o dorso do pé contra a borda de uma superfície plana. Teste cada lado separadamente. \| Sem resposta  D E \| \| Somente dorsiflexão do tornozelo  D E \| Total resposta de colocação com flexão do quadril, joelho e colocação plantar sobre a superfície  D E \|  \|  \| \| Incidência \| 1% \| \| 18% \| 81% \|  \|  \| \| **Figura 21** – Colocação. Uma resposta completa de colocação com flexão dos quadris, joelhos e tornozelos (coluna 3) e uma resposta de colocação com somente flexão do tornozelo foram consideradas ótimas (coluna 2). A ausência de resposta foi considerada subótima.  **Movimentos (Figura 22-24)** \| \| \| \| \| \| \| \|  \| Coluna 1 \| \| Coluna 2 \| Coluna 3 \| Coluna 4 \| Coluna 5 \| \| Movimentos espontâneos (quantitativo): observe o lactente deitado na posição supina. \| Sem movimento \| \| Movimentos curtos e esporádicos isolados \| Movimentos frequentes isolados \| Movimentos generalizados frequentes \| Movimentos contínuos exagerados \| \| Incidência \| <1% \| \| 3% \| 5% \| 92% \| <1% \| \| **Figura 22** – Movimentos espontâneos (quantitativo). Movimentos generalizados e alternados frequentes. (coluna 4) foram considerados ótimos. Movimentos isolados frequentes (coluna 3) foram considerados limítrofes e todos os outros padrões foram classificados como subótimos. \| \| \| \| \| \| \| \|  \| Coluna 1 \| \| Coluna 2 \| Coluna 3 \| Coluna 4 \| Coluna 5 \| \| Movimentos espontâneos (qualitativo): observe o lactente deitado na posição supina \| Somente alongamento \| \| Alongamentos frequentes e movimentos abruptos, alguns movimentos suaves também estão presentes \| Movimentos fluentes, mas monótonos \| Movimentos alternados fluentes de braços + pernas, boa variabilidade \| Clônus sincronizados, caretas, espasmos e outros movimentos anormais \| \| Incidência \| 2% \| \| 5% \| <1% \| 93% \| <1% \| \| **Figura 23** – Movimentos espontâneos (qualitativo). Movimentos alternados fluentes (coluna 4) com boa variabilidade foram considerados ótimos. Alongamentos frequentes, alternados com movimentos fluentes (coluna 2) foram considerados como limítrofes, e todos os outros padrões foram classificados como subótimos. \| \| \| \| \| \| \| \|  \| Coluna 1 \| \| Coluna 2 \| Coluna 3 \| Coluna 4 \| Coluna 5 \| \| Elevação da cabeça em posição prona: Lactente posicionado em posição prona cabeça na linha média \| Sem resposta \| \| Lactente vira a cabeça, queixo não se eleva \| Lactente vira a cabeça, queixo se eleva \| Lactente eleva a cabeça e o queixo \| Lactente eleva a cabeça e a mantém \| \| Incidência \| <1% \| \| 10% \| 50% \| 40% \| <1% \| \| **Figura 24** – Elevação da cabeça (prona). Quando os lactentes foram posicionados em prona, a elevação do queixo com a cabeça virando para o lado (coluna 3) ou a cabeça totalmente elevada (coluna 4) foi considerada ótima. Virar a cabeça sem o queixo elevar-se foi considerado limítrofe e todos os outros padrões foram classificados como subótimos.  **Sinais Anormais (Figura 25-27)** \| \| \| \| \| \| \| \|  \| Coluna 1 \| \| Coluna 2 \| Coluna 3 \| Coluna 4 \| Coluna 5 \| \| Postura anormal das mãos ou dos dedos dos pés. \|  \| \| Mão abertas, dedos dos pés estendidos na maior parte do tempo \| Mãos cerradas intermitentes ou adução do polegar \| Mãos cerradas continuamente ou adução do polegar, flexão do indicador, oposição do polegar \| Extensão contínua dos dedões ou flexão de todos os dedos do pé \| \| Incidência \|  \| \| 85% \| 12% \| 3% \| <1% \| \| **Figura 25** – Postura anormal das mãos e dedos dos pés. Mãos abertas e postura normal dos dedos dos pés (coluna 2) ou mãos cerradas intermitentes e adução do polegar. (coluna 3) foi considerada ótima. Todos os outros padrões foram classificados como subótimos. \| \| \| \| \| \| \| \|  \| Coluna 1 \| \| Coluna 2 \| Coluna 3 \| Coluna 4 \| Coluna 5 \| \| Tremor \|  \| \| Sem tremor ou tremor apenas quando chora \| Tremor somente após reflexo de Moro ou ocasionalmente quando acordado \| Tremor frequente quando acordado \| Tremor contínuo \| \| Incidência \|  \| \| 88% \| 12% \| <1% \| <1% \| \| **Figura 26** – Tremores. Sem tremor, tremor somente quando chora (coluna 2) ou tremor apenas após reflexo de Moro , e tremores ocasionais quando acordado (coluna 3) foram considerados ótimos. Todos os outros padrões foram classificados como subótimos. \| \| \| \| \| \| \| \|  \| Coluna 1 \| \| Coluna 2 \| Coluna 3 \| Coluna 4 \| Coluna 5 \| \| Susto \| Sem susto mesmo com barulho inesperado \| \| Sem sustos espontâneos, mas reage à barulhos inesperados \| 2-3 sustos espontâneos \| Mais de 3 sustos espontâneos \| Sustos contínuos \| \| Incidência \| <1% \| \| 94% \| 6% \| <1% \| <1% \| \| **Figura 27** – Sustos. Ausência de sustos espontâneos (coluna 2) ou 2 ou 3 sustos espontâneos (coluna 3) foram considerados ótimos. Todos os outros padrões foram classificados como subótimos.  **Comportamento (Figura 28-34)** \| \| \| \| \| \| \| \|  \| Coluna 1 \| \| Coluna 2 \| Coluna 3 \| Coluna 4 \| Coluna 5 \| \| Movimento dos olhos \| Não abre os olhos \| \|  \| Movimento dos olhos totalmente conjugados \| Movimentos transitórios: nistagmo, estrabismo, movimentos vagos dos olhos, sinal do sol poente \| Movimento persistente: nistagmo, estrabismo, movimentos vagos dos olhos, sinal do sol poente \| \| Incidência \| 7% \| \|  \| 92% \| 1% \| <1% \| \| **Figura 28** – Movimento dos olhos. A abertura dos olhos (coluna 1) não pôde ser provocada em 7%. Os movimentos oculares simétricos normais (coluna 3) foram considerados ótimos. Todos os outros padrões foram classificados como subótimos. \| \| \| \| \| \| \| \|  \| Coluna 1 \| \| Coluna 2 \| Coluna 3 \| Coluna 4 \| Coluna 5 \| \| Orientação auditiva: Lactente acordado, enrole-o. Segure o chocalho entre 10 a 15 cm da orelha \| Sem reação \| \| Susto auditivo, anima-se e fixa, mas sem uma orientação \| Os olhos se movem, a cabeça pode virar em direção à fonte \| Vira a cabeça de forma prolongada ao estímulo, procura suavemente com os olhos \| Vira cabeça e olhos para o barulho todas às vezes, espasmódico, abrupto \| \| Incidência \| <1% \| \| 30% \| 50% \| 20% \| <1% \| \| **Figura 29** – Orientação auditiva. Olhos e cabeça virando para o lado do barulho (colunas 3 e 4) ou uma resposta mais fraca (animar-se ou virar a cabeça com os olhos fechados). (coluna 2) foi considerado ótimo. Todos os outros padrões foram classificados como subótimos. \| \| \| \| \| \| \| \|  \| \| Coluna 1 \| Coluna 2 \| Coluna 3 \| Coluna 4 \| Coluna 5 \| \| Orientação visual: Enrole o lactente, acorde-o com o chocalho se necessário, ou balance delicadamente. Observe se o lactente pode ver e seguir a bola vermelha (B) ou o alvo (A) \| \| Não segue ou foca o estímulo  B A \| Fixa, foca, segue brevemente para o lado, mas perde o estímulo  B A \| Segue horizontal e verticalmente, não vira a cabeça  B A \| Segue horizontalmente e verticalmente, vira a cabeça  B A \| Segue em círculo  B A \| \| Incidência \| \| <1% \| 7% \| 41% \| 51% \| 1% \| \| **Figura 30** – Atenção visual. 7% dos lactentes tinham seus olhos fechados ao longo do exame e não puderam ser testados. A habilidade de seguir verticalmente ou horizontalmente com e sem movimento da cabeça (colunas 3 e 4) foram considerados ótimos. Seguir incompletamente foi considerado limítrofe e todos os outros padrões foram classificados como subótimos. \| \| \| \| \| \| \| \|  \| Coluna 1 \| \| Coluna 2 \| Coluna 3 \| Coluna 4 \| Coluna 5 \| \| Atenção: Testada como resposta a estímulo visual (bola vermelha ou alvo) \| Não responde a estímulos \| \| Quando acordado, olha somente brevemente \| Quando acordado, olha para o estímulo, mas o perde \| Mantém interesse no estímulo \| Não se cansa (hiper-reativo) \| \| Incidência \| 1% \| \| 2% \| 38% \| 49% \| <1% \| \| **Figura 31** – Atenção. A atenção foi testada como a qualidade da resposta dos lactentes para seguir os estímulos apresentados (uma bola vermelha ou um alvo). Olhar para o estímulo com interesse breve (coluna 3) ou prolongado (coluna 4) foi considerado ótimo. Todos os outros padrões foram classificados como subótimos. \| \| \| \| \| \| \| \|  \| Coluna 1 \| \| Coluna 2 \| Coluna 3 \| Coluna 4 \| Coluna 5 \| \| Irritabilidade: Em resposta ao estímulo \| Quieto todo o tempo, não se irrita aos estímulos \| \| Acordado, chora às vezes quando manipulado \| Chora frequentemente quando manipulado \| Chora sempre quando manipulado \| Chora mesmo quando não é manipulado \| \| Incidência \| <1% \| \| 93% \| 5% \| 2% \| <1% \| \| **Figura 32** – Irritabilidade. Choro ocasional quando manipulado (coluna 2) foi considerado ótimo. Choro frequente quando manuseado (coluna 3) foi considerado limítrofe e todos os outros padrões foram classificados como subótimos. \| \| \| \| \| \| \| \|  \| Coluna 1 \| \| Coluna 2 \| Coluna 3 \| Coluna 4 \| Coluna 5 \| \| Choro \| Não chora \| \| Apenas choraminga \| Chora ao estímulo, mas com intensidade normal \|  \| Chora de forma intensa, e frequentemente contínua \| \| Incidência \| <1% \| \| 7% \| 92% \|  \| 1% \| \| **Figura 33** – Choro. Choro normal em resposta ao estímulo (coluna 3)foi considerado ótimo. Choramingar foi classificado como limítrofe, e todos os outros padrões foram classificados como subótimos. \| \| \| \| \| \| \| \|  \| Coluna 1 \| \| Coluna 2 \| Coluna 3 \| Coluna 4 \| Coluna 5 \| \| Consolabilidade: Facilidade para acalmar o lactente. \| Adormecido ou acordado, não chora, não necessita consolo \| \| Acordado, chora brevemente, não necessita consolo \| Acordado, chora, se acalma quando fala com ele \| Acordado, chora, necessita de colo para consolar \| Acordado, chora, inconsolável \| \| Incidência \| 1% \| \| 41% \| 45% \| 12% \| 1% \| \| **Figura 34** – Consolabilidade. Lactentes que não estavam chorando ou chorando brevemente e não precisaram de consolo, puderam ser consolados pela fala ou precisaram de colo para consolar foram considerados ótimos. ( colunas 2, 3 e 4) foram considerados ótimos. Todos os outros padrões foram classificados como subótimos. \| \| \| \| \| \| \| | | | | | |


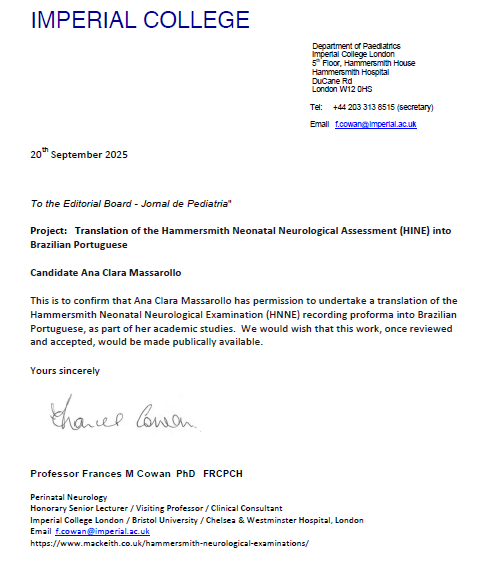

Supplement: Supplementary file 1 [file mmc1.docx]
